# COVID-19 Physician Burnout: Louisiana's Workforce Vulnerability and Strategies for Mitigation

**DOI:** 10.31486/toj.22.0072

**Published:** 2023

**Authors:** Richard Shane Barton, Tara Saxena, Carver Montgomery, Denise Bates-Fredi, Matthew Kelley, Patrick A. Massey

**Affiliations:** ^1^ Department of Orthopaedic Surgery, Louisiana State University Health–Shreveport, Shreveport, LA; ^2^ Department of Public Health, Louisiana State University Health–Shreveport, Shreveport, LA; ^3^ Department of Kinesiology and Health Science, Louisiana State University Health–Shreveport, Shreveport, LA

**Keywords:** *Coronavirus*, *pandemics*, *public health*

## Abstract

**Background:** Louisiana is historically one of the lowest-performing states in terms of health outcomes in the United States. Hurricane Katrina led to a decrease in available health care resources, with a larger impact on resources for those living below the poverty line. Subsequently, the coronavirus disease 2019 (COVID-19) pandemic has been shown to have had disproportionately large impacts on minority communities, uninsured populations, and rural communities—all of which are found in Louisiana.

**Methods:** This review focuses on the unique challenges of health care in Louisiana, the influence of COVID-19 on physician burnout, and methods of reducing work exhaustion for those in the health care field.

**Results:** A national survey showed that physician satisfaction decreased from June 29, 2021, through September 26, 2021, compared to before the pandemic. A critical component in the provision of the essential services of public health is the ability to build and sustain a clinically skilled and diverse physician workforce. Maintaining well-being and retaining the physician workforce are prerequisites to the equitable provision of access to health care services.

**Conclusion:** Maintaining one's own wellness is critical to occupational sustainability, particularly when unique stressors such as those encountered during the COVID-19 pandemic are present. The future of a vital health care system depends on physicians maintaining healthy habits and seeking help when burnout symptoms are recognized, both at the individual and institutional level.

## LOUISIANA DEMOGRAPHICS AND HEALTH BENCHMARKS

**Figure 1.**
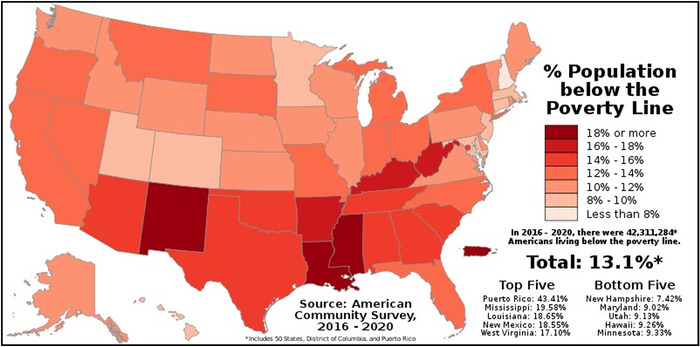
**Poverty by state in the United States. Louisiana is among the top 5 states and territories with the highest percentage of the population living in poverty.^3^** (Image reproduced without changes according to the provisions of the CC BY-SA 4.0 license.)

Louisiana is the 25th most populous state in the United States. According to 2020 US Census data, the population estimate for the state on July 1, 2021 was almost 4,625,000.^1^ The racial makeup of the population is 62.4% White (not Hispanic or Latino), 33.0% Black or African American, 5.6% Hispanic or Latino, 1.9% Asian, 0.8% American Indian and Alaska Native, 0.1% Native Hawaiian and other Pacific Islander, and 1.8% reporting 2 or more races.^1^ In 2014, only 3 of the state's 64 parishes (counties) had more than 300,000 nonelderly residents, indicating a significant concentration of the population in only a few metropolitan areas.^2^ The remainder of the state is generally considered rural. More than 23% of Louisiana residents are children, and almost 86% of the population aged 25 years or older has at least a high school degree.^1^ In 2014, approximately 22% of nonelderly residents had at least a college degree, and 12% of people were aged 65 years or older.^2^ The median household income in the period 2016-2020 was $50,800.^1^ People living in poverty account for almost 20% of the population (as illustrated in Figure 1)^3^ vs the US national average of 15%.^2^ In 2014, 33% of Louisiana children lived in poverty. Black people are almost three times more likely than White people to live in poverty.^2^

Louisiana has a history of poor performance on national health benchmark surveys, ranking between 47th and 50th from 2009 to 2019 on numerous national surveys.^4-6^ However, improvements have been noted during the past few years, with Louisiana moving up 6 positions from 50th in 2014 to 44th in 2020 for overall performance. The greatest improvement was noted in addressing health care disparity as Louisiana moved up 15 positions to 28th.^7^

## IMPACTS OF HURRICANE KATRINA AND THE AFFORDABLE CARE ACT ON LOUISIANA PUBLIC HEALTH

Hurricane Katrina, which occurred on August 29, 2005, placed a tremendous burden on the infrastructure of the health delivery system and disaster response network in Louisiana.^8^ More than 1.1 million residents were forced to leave their homes in the New Orleans area. This exodus affected not only Louisiana but many neighboring states. Factors that impact access to health care such as health care coverage and the availability of health care providers are of critical importance, and these shortages were exacerbated by the hurricane.^2^ Drinking water and sanitation systems were disrupted, and routine care for chronic conditions was in limited supply for years. Only recently has Louisiana begun to recover from the repercussions of this disaster.^4^ Charity Hospital, previously the primary safety-net hospital for the New Orleans region, was permanently closed after Hurricane Katrina, with a redistribution of these services to numerous other health care facilities.^9^ The University Medical Center complex has since been developed and is filling much of that need now, but the effects of Hurricane Katrina left long-term changes in the delivery of health care for a vulnerable population of poor, elderly, and chronically ill patients.

The Affordable Care Act of 2010, while still debated among policymakers and politicians, has had a positive effect on the number of Louisiana residents who were previously uninsured. Uninsured rates declined by 6.3% between 2013 and 2016,^10^ in contrast to early findings in 2014 and 2015 showing that much of the Louisiana population fell into the coverage gap before Medicaid expansion was fully implemented in 2016. By 2018, Louisiana's uninsured population was 8%, half of the level of 16.6% immediately prior to expansion.^10^ While some improvements have been made, Louisiana still had an adult uninsured rate of 9.4% in 2021 and a Medicaid rate of 20%.^11^ In response to the coronavirus disease 2019 (COVID-19) pandemic, the 117th US Congress passed the American Rescue Plan of 2021 (COVID-19 Stimulus Package) that was signed into law on March 11, 2021. Although $315 million was appropriated to Louisiana as part of this package, the majority of the money was allocated to the Water Sector Program for sewer and water repairs.^12^

## PHYSICIAN SUPPLY

**Figure 2.**
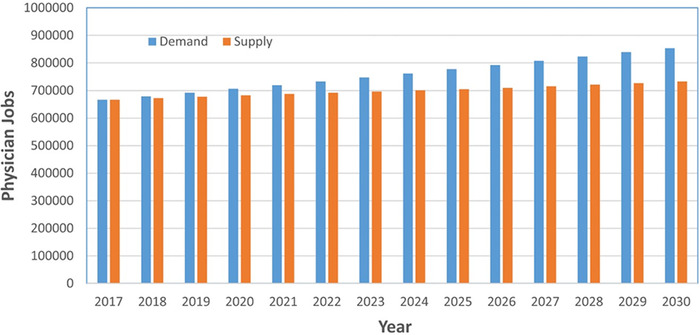
**Projected US physician demand and supply from 2017-2030. Using publicly available databases, Zhang et al created this model illustrating the probable increased demand for physicians compared to the proposed supply until 2030.^14^** (Image reproduced without changes according to the provisions of the CC BY 4.0 license.)

The Association of American Medical Colleges (AAMC) tracks the predicted physician supply, as well as the supply of physician assistants and nurse practitioners. The latest report, released in June 2021, projects a nationwide shortage of up to 48,000 primary care physicians and up to 77,100 specialty physicians by the year 2034.^13^ The report cites numerous reasons, including a growing and aging population, with a 42% increase in the American population older than 65 years by 2034. Two of 5 practicing physicians are predicted to be age 65 years or older by 2034. The need to make health care more accessible to a broader population depends on numerous factors, and physician supply is a major determinant of access, but improving access and population health increases the demand for physicians.^13^ Figure 2 depicts an estimation from Zhang et al of the increased demand of physicians compared to the supply for the years 2017 through 2030.^14^

## COVID-19 AND PHYSICIAN BURNOUT

COVID-19, caused by the severe acute respiratory syndrome coronavirus 2, was declared a global pandemic by the World Health Organization on March 11, 2020.^15^ The Johns Hopkins Coronavirus Resource Center reported that as of June 19, 2021, almost 180,000,000 global cases had been confirmed, with more than 33,500,000 in the United States.^16^ At that time, the global death toll had reached more than 3,850,000 lives lost, with the United States surpassing 601,000 deaths.^16^ The numbers have increased substantially since then.

The COVID-19 pandemic affected people worldwide, with health care providers facing unprecedented challenges in their ability to provide care for patients. This challenging time was marked by personal protective equipment shortages, facility closures, and supply chain collapse, with patients often delaying care out of fear of contracting the disease. In addition to these issues were the personal challenges of managing stressed home situations, with the illnesses and deaths of loved ones, school closures, and job loss for many family members. These stresses can lead to increased risks of physician burnout and decreased wellness, topics that were the subject of scrutiny even prior to the pandemic.^17^

Prior to COVID-19, more than 40% of physicians reported symptoms of burnout at least once a week.^18^ The unique stressors of the pandemic have had a significant negative impact on the well-being of physicians.^19^ Physicians not only worry about the health of their patients but also about getting sick themselves or exposing their colleagues, families, and friends to the potentially deadly virus.

**Figure 3.**
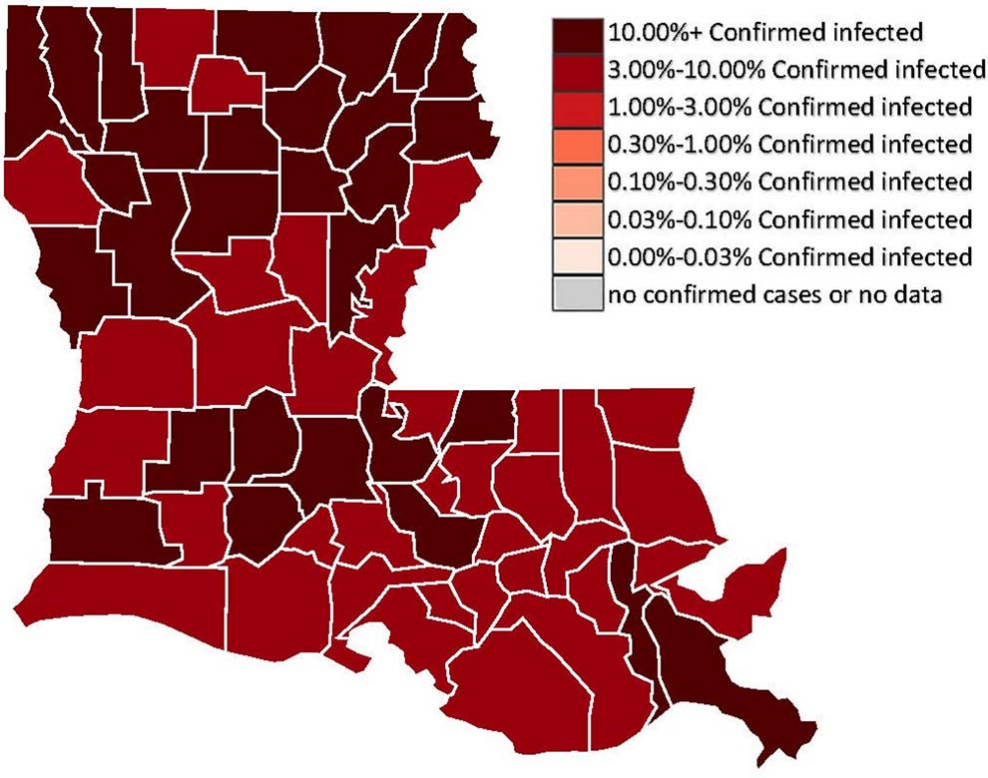
**Coronavirus disease 2019 (COVID-19) prevalence in Louisiana as of March 21, 2021, when at least 1% of the population of every parish (county) had confirmed COVID-19 infection.^20^** (Image reproduced without changes according to the provisions of the CC0 1.0 license.)

The virus has been shown to have disproportionately large impacts on minority communities, uninsured populations, and rural communities—all of which are found in Louisiana.^2^ Louisiana's physician workforce and safety-net hospitals play a critical role in the delivery of health care to the most vulnerable populations. Figure 3 shows just how prevalent COVID-19 infections were in the state of Louisiana in early 2021, with every parish having at least 1% of its population with a confirmed case.^20^ With so much of the population affected by the virus, it is not surprising that a 2021 financial analysis ranked Louisiana as the state with the hardest-hit economy in the nation after COVID-19.^5^ The vulnerabilities of health care infrastructures worldwide were exposed by the strain placed on them by the surge of acutely ill patients entering the health care system due to COVID-19. These impacts were felt disproportionately by poor and minority populations, particularly in Louisiana.^9^ Long-term care, acute hospital care, and mental health care suffered because of the effect of the pandemic on the already struggling Louisiana health care system.^21^

Prior to the global pandemic, the adverse effect of burnout on physician wellness had become a target for intervention to improve physician well-being and health care outcomes for their patients.^22^ The unique stressors associated with COVID-19 have added to and exacerbated preexisting causes of physician burnout, placing a large psychological burden on the health care workforce.^23^ A 2022 Medscape survey found that 81% of physicians were “somewhat” or “very” happy outside of work prior to the pandemic, compared to 59% during the pandemic (the time period of June 29, 2021, through September 26, 2021).^24^ This decrease in satisfaction is in addition to the anticipated physician shortage predicted by the AAMC.^13^ A substantial portion of Louisiana's population becomes disproportionately vulnerable as resources, such as the physician workforce, become even scarcer.^25^ Considering physicians’ role in the provision of public health services, the effect of burnout on physician wellness, and the potential impact of COVID-19 on health care providers in Louisiana is critical to creating a sustainable physician workforce, as physicians experiencing burnout are more likely to leave the field of medicine.^26^

## FACTORS ASSOCIATED WITH BURNOUT

The body of literature on physician burnout increased from approximately 1,000 publications in 2002 to approximately 17,000 publications in 2021.^27^ Several studies have examined the self-efficacy theory of health behavior and evaluated its relationship to physician burnout.^28-30^ Self-efficacy is one's belief in their own capabilities to take action to complete a specific goal. These studies consistently reported a negative strong relationship between self-efficacy and burnout.^28-30^ In other words, the higher someone is rated for self-efficacy, the less likely the individual is to experience burnout. In their survey of 177 surgeons, Janko and Smeds showed that self-efficacy and social support decreased burnout.^29^ Smeds et al showed that in addition to self-efficacy, mentorship was also important for decreasing burnout.^28^ A survey by Milam et al of 179 surgery residents showed that gender was not associated with self-efficacy, but self-efficacy was a major contributor to physician well-being.^30^

A review of the literature suggests that physicians’ greatest health need is related to the difficulty of balancing work (clinical service, academic pressures, long hours) with relationships (spouses, children, extended family, friends). This difficulty can lead to burnout and a subsequent cascade into a series of detrimental conditions that put the health and well-being of the resident or attending physician, their family and friends, and their patients at risk.^31^

Sargent et al conducted a national survey of orthopedic residents, faculty members, and resident and faculty spouses to assess coping mechanisms, job satisfaction, demographic information, stress, and perceptions of harassment.^32^ Published in 2009, the study reported high levels of burnout in more than half of the resident respondents, almost 30% of the faculty, and 13% and 30% of the faculty and resident spouses, respectively. In a follow-up paper published in 2011, Sargent et al reported that female residents in their second year of postgraduate training were at the greatest risk of burnout. Risks associated with the impaired physician include depression, burnout, substance abuse, and disruptive behavior.^31^

Noting that physicians are at risk of burnout, Sargent et al noted, “An extraordinary intellect, education, work ethic, and capacity for stamina under stress will not make you immune to the wear and tear effects of a mismanaged lifestyle.”^31^ The importance of finding meaning in work and life is critical. Resilience, the ability to persevere through times of change or uncertainty, is a critical component of successfully coping with burnout. The importance of family and emotional and social outlets is reinforced in both papers by Sargent et al.^31,32^

The provision of essential public health services is dependent on a resilient and sustainable health care workforce. If left unaddressed, the impact of burnout on physician attrition will be more severe in the future, as a Medical Group Management Association Stat poll showed that 41% of physicians who unexpectedly retired in the prior year stated their reason was related to the COVID-19 pandemic (burnout, health risks, loss of reimbursement).^33^

## GOING FORWARD: STRATEGIES TO REDUCE BURNOUT

**Figure 4.**
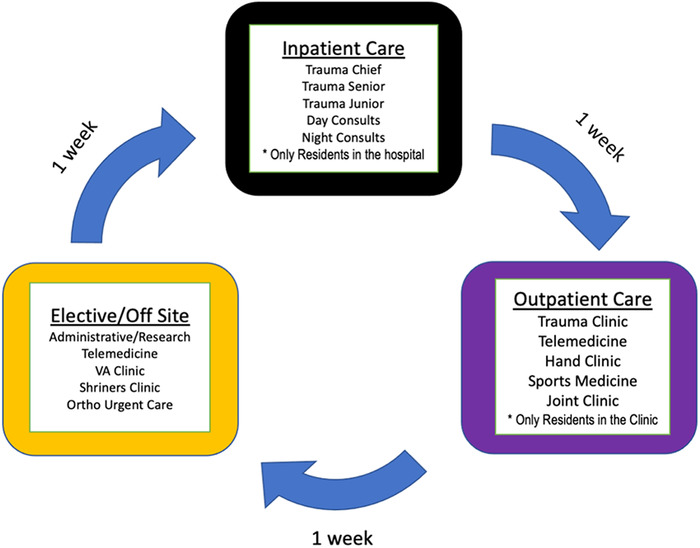
**Restructured schedule proposed by Zhang et al for orthopedic residents to reduce the chance of coronavirus disease 2019 (COVID-19) exposure. The schedule rotates every 7 days so that each team reduces the chances of spreading COVID-19 to their colleagues and the general population.^35^** (Image reproduced without changes according to the provisions of the CC BY-NC-ND 4.0 license.) VA, Veterans Administration.

The impact of burnout and wellness on physician supply is a concern to be addressed now. Physicians must be encouraged by the medical profession to address their own overall health to sustain their availability to provide care.^34^ Excessive work hours and lack of sleep for both practicing physicians and residents must be addressed. Zhang and colleagues described evidence-based interventions for orthopedic surgery training programs that provide meaningful education to trainees while maintaining high-quality patient care. One example is restructuring rotations with dedicated work teams to limit physicians’ and residents’ exposure to COVID-19. These rotations include a 3-team approach, with 1 week in the acute care setting where exposure risk is greatest, followed by 2-week rotations away from the inpatient setting (Figure 4).^35^ Massey et al described the creation of surgical paradigms to best utilize limited resources.^36^ Elective surgical case volumes were limited to conserve personal protective equipment and limit physician and patient exposures to high-density COVID-19 environments to the extent possible, including using telemedicine to a greater extent than before the pandemic to deliver health care in a low-risk environment.^36^

While decreasing hours and work burden can decrease burnout, other factors may also be beneficial. As previously mentioned, self-efficacy is a major protector against burnout. Providing resources to empower physicians so that they can achieve their goals may improve their self-efficacy.^30^ Additionally, providing social events and dedicated mentorship may also be useful strategies for decreasing burnout.^29^ Implementation of exercise programs should also be an essential element of improving wellness and decreasing burnout.^37^

Hooper and colleagues reported on effective techniques to address the psychological impact of COVID-19 on health care workers.^23^ These techniques include psychological first aid, eye movement desensitization and reprocessing, resilience at work programs, mindfulness, and trauma risk management. Trauma risk management techniques helped to reduce the stigma and barriers to physicians seeking assistance when they began to suffer from the symptoms of burnout.^24^

## CONCLUSION

Maintaining one's own wellness is critical to occupational sustainability, especially when unique stressors such as those encountered during the COVID-19 pandemic are present. The future of a vital health care system depends on physicians maintaining healthy habits and seeking help when burnout symptoms are recognized, both at the individual and institutional level. The downstream impacts on public health of a relatively decreased physician workforce coupled with increased physician demand are tremendous. These impacts are even more apparent in health care settings associated with poverty, unemployment, and lack of adequate insurance.

## Notes

*This article meets the Accreditation Council for Graduate Medical Education and the American Board of Medical Specialties Maintenance of Certification competencies for Medical Knowledge, Professionalism, and Practice-Based Learning and Improvement.*
